# The influence of personality traits on university performance: Evidence from Italian freshmen students

**DOI:** 10.1371/journal.pone.0258586

**Published:** 2021-11-03

**Authors:** Luca Corazzini, Silvia D’Arrigo, Emanuele Millemaci, Pietro Navarra

**Affiliations:** 1 Department of Economics and VERA, University “Ca’ Foscari”, Venezia, Italy; 2 Department of Economics, University of Messina, Messina, Italy; Aalborg University, DENMARK

## Abstract

Despite several attempts to provide a definite pattern regarding the effects of personality traits on performance in higher education, the debate over the nature of the relationship is far from being conclusive. The use of different subject pools and sample sizes, as well as the use of identification strategies that either do not adequately account for selection bias or are unable to establish causality between measures of academic performance and noncognitive skills, are possible sources of heterogeneity. This paper investigates the impact of the Big Five traits, as measured before the beginning of the academic year, on the grade point average achieved in the first year after the enrolment, taking advantage of a unique and large dataset from a cohort of Italian students in all undergraduate programs containing detailed information on student and parental characteristics. Relying on a robust strategy to credibly satisfy the conditional independence assumption, we find that higher levels of conscientiousness and openness to experience positively affect student score.

## Introduction

In a seminal study, Borghans et al. [1] propose a conceptualization of personality traits as skills that make an independent contribution to the map of individuals’ preferences, choices and behaviours compared with the conventional measures of mental or cognitive skills adopted by economists. While foremost economic literature has thoroughly examined the role of cognitive functioning to learn, process and store knowledge in explaining socioeconomic success, more recently, economists have started focusing on metrics of personality endowments using the theoretical frames defined by personality psychologists. Educational investments in noncognitive skills that the labour market rewards can contribute to explain the connection between schooling and returns to earnings [2]. Previous literature suggests that personality traits affect labour market outcomes including occupational attainment and individual earnings [3–7], possibly through their impact on productivity [8–10], and the related effects on the probability of finding a job and the duration of unemployment [11, 12]. Personality traits can influence the way people respond to stressful conditions in competitive settings and likely produce consequences on performance in real-world scenarios [13]. In addition, while cognitive abilities show a critical impact on achievement test scores and educational attainment, personality traits produce distinct incentives to knowledge acquisition [14, 15].

While remaining relatively stable in adulthood, personality traits observe a steady process of maturation in childhood and adolescence, during which public policy interventions are impressive and have long-term consequences (see Almlund et al. [16] and Kautz et al. [17] for a detailed discussion about the possible mechanisms of development and change of personality traits).

Findings on the effects of personality traits on economic and other life outcomes are stimulating policy makers in better designing educational public policies for improving student achievement and adult outcomes, especially through early intervention strategies that aim at influencing the noncognitive skill formation [18–20].

Improving quality of learning and educational attainment to meet the increasing demand for high-level skills in the labour market are challenging objectives of public policies. As strategic elements of its political agenda for economic growth, in 2010, the European Union (EU) set the targets of improving tertiary education attainment and reducing early school leavers by 2020. Albeit the proportion of 25–34 years old individuals with a tertiary level of education increased by 9 percentage points on average across OECD countries in the decade 2008–2018 [21], structural discrepancies in performance persist. In this respect, Italy appears to be one of the most problematic countries. For instance, the percentage of Italian population aged 30–34 who had successfully completed tertiary studies was only 27.6 against the EU average of 40.3 in 2019, while school dropout rates amounted to 13.5 per cent (EU 10.2) of the population in the range 18–24 [22, 23]. Moreover, the number of young people aged 15–34 who started but never completed tertiary education, according to the most recent data, was more than half a million, far above the EU average [24]. Since earlier investments in human capital beget skills at later stages of life in the dynamic process of learning [25], educational programmes fostering noncognitive skills can provide school-age children with valuable resources to pursue a successful student career. Accordingly, since personality traits or psychological styles can affect the probability of dropping out from school, studies analysing the psychological profiles of weaker students can support appropriate public strategies for addressing the issue [26]. The pivotal nature of the connection between noncognitive skills and increases in education can provide some guidance to reduce the divergent trajectories of national education systems.

The present study focuses on the personality-related determinants of performance in post-secondary education. The empirical analysis takes advantage of a unique dataset from a cohort of 3,242 freshmen students aged between 18–24 years that in 2016 entered the degree programmes of the University of Messina, a large and public Italian university. The interest of this paper is in estimating the impact of self-reported scales of personality traits on the grade point average (GPA), a measure of the exams successfully passed at the end of the first year of courses. Personality assessment relates to the Five-Factor Model, an internationally well-established framework of personality traits, based on the following broad dimensions or *Big Five*: extraversion, agreeableness, conscientiousness, emotional stability (also referred to as the opposite pole of neuroticism) and openness to experience. At the time of matriculation, prospective students are required to provide answers to the Italian adaptation of the Ten-Item Personality Inventory [27], a popular personality questionnaire to infer measures of the Big Five traits [28].

The results indicate that higher scores on conscientiousness and openness to experience have a positive impact on GPA. The estimates suggest that these Big Five traits affect academic achievement besides the human capital background acquired through schooling. Parental education, occupational status and industry are not responsible for the estimated effect of personality traits on academic performance. We find significant gender differences in performance, meaning that women outperform men in GPA. Albeit the density distributions of the Big Five trait scores—except for openness to experience—significantly diverge between men and women, we do not find heterogenous effects of personality traits on GPA by gender.

Previous studies focusing on the predictive value of the Big Five traits on performance in higher education reported mixed results. This paper addresses a series of limitations that may explain such inconclusive evidence.

First, unlike earlier research, the data is not restricted to students from one or more majors and does not depend on the limitations imposed by a small sample size, given that all freshmen participated in the survey. The use of comprehensive data on student population makes our findings generalizable to freshmen students of similar higher institutions, instead of being driven by the characteristics of specific student groups. Of course, the results of this study do not apply to the non-student population even though in the same age group. However, it is noteworthy that student population is the sample of interest for education research aiming at establishing the determinants of academic achievement in higher education conditionally on attending a university course.

Second, a simple OLS estimation does not avoid from the risk that the estimated effect of the personality traits on academic performance is driven by endogeneity in particular in two specific ways: i) the observed outcomes could reflect an omitted variable bias, if personality traits are associated with unobservable indices that influence the dependent variable; ii) or reverse causality, if the test scores affect personality traits. This is an important point for research since the inference of causal effects in personality traits has direct implications for the evaluation of public policies to augment student achievement. First of all, the empirical strategy of this paper relies on OLS estimations where the GPA is regressed on, apart from the personality traits variables, an ample set of predetermined characteristics—i.e., neither choice variables nor events occurred after personality measurement- drawn from a comprehensive review of the literature, and that might correlate with both Big Five traits and GPA. A battery of tests provides support in favour of the reliability of the chosen set of variables and the outcome of the regressions, provided that the tests proposed by Bellows and Miguel [29] and Oster [30] suggest that the estimated effects cannot entirely be attributed to a bias driven by the relationship between personality traits and unobservable components in the regression model (selection on unobservables). Moreover, previous literature extensively relied on personality questionnaires administered during students’ classes. Since personality traits in this study were collected when undergraduate students enrolled in their first year of university, that is a time in which they had no a priori knowledge about course attendance and test scores, the results do not seem to be affected by a reverse causality bias.

Third, although the distribution of personality traits across areas of study is driven by a selection process (i.e., the enrolment decision), we show that personality traits can be considered as randomly assigned across areas of study after controlling for the predetermined characteristics in the regression equation.

The remainder of the paper is structured as follows. The next section reviews the existing literature on the relationship between the Big Five personality traits and performance in higher education. Sections 3 and 4 present data and empirical strategy, respectively. Section 5 discusses the results and the last section concludes.

## Overview of the existing literature

Personality traits are the “relatively enduring patterns of thoughts, feelings, and behaviors” [31] that, interactively with cognitive processes, motivational aspects and beliefs, reflect adaptive responses to external conditions. The Big Five Factor structure, based on a set of broad psychological constructs that hierarchically encompass narrower qualities (or facets), has gained increasing popularity in the last decades and is largely accepted as the most influential taxonomy of personality traits [1]. Table 1 summarizes the main characteristics and findings of relevant and influential studies that examine the relationship between the Big Five traits and academic examination grades in higher education.

**Table 1:** Previous findings on the effects of the Big Five personality traits on post-secondary examination grades.

Study	Sample	Method	Controls	E	A	C	ES	O
Burks et al. (2015) [32]	Undergraduate students in liberal arts (n = 100), US	Tobit model	Age, race, sex, family income, time preference, risk aversion, cognitive abilities (non-verbal IQ, numeracy, planning ability)	0	0	+	0	0
Chamorro-Premuzic & Furnham (2003) [33]	Undergraduate students in psychology (n = 247), GBR	Correlation analysis/ Multiple regression	None	0/-	0	+	+/0	0
Chamorro-Premuzic & Furnham (2003)—sample 1 [34]	Undergraduate students in psychology (n = 70), GBR	Correlation analysis/ Multiple regression	None	0	0	+	+	0
Conard (2006) [35]	Undergraduate students in psychology (n = 289), US	Correlation analysis	None	0	0	+	0	0
	Undergraduate students in psychology (subsample of n = 186), US	Hierarchical regression analysis	Class attendance, SAT	0	0	+	0	0
Diseth (2003) [36]								
sample 1	Undergraduate students in psychology (n = 127), NO	Correlation analysis	None	0	0	0	0	0
sample 2	*Examen philosophicum* students (n = 101), NO	Correlation analysis	None	0	-	0	-	+
Farsides & Woodfield (2003) [37]	Undergraduate students (n = 432), GBR	Correlation analysis/ Hierarchical regression analysis	None/Cognitive abilities (verbal IQ, spatial IQ), seminar attendance, non-assessed work submission indicators	0	+/0	0	0	+
Furnham et al. (2003) [38]	Undergraduate students in psychology (n = 93), GBR	Correlation analysis/ Hierarchical regression analysis	None/Gender, beliefs about intelligence (BAI), general cognitive ability (WPT)	-	0	+	0	0
Gray & Watson (2002) [39]	Undergraduate students (n = 300), US	Correlation analysis	None	0	+	+	0	+
Lounsbury et al. (2003) [40]	Undergraduate students in psychology (n = 175), US	Correlation analysis	None	0	0	+	0	+
Kappe & van der Flier (2012) [41]	Undergraduate students in professional school of human resource management (n = 137), NL	Multiple regression analysis	Intelligence, intrinsic motivation, anxiety, need for pressure, need for status, study motivation	0	0	+	0	0
Komarraju et al. (2009) [42]	Undergraduate students (n = 308), US	Correlation analysis/ Multiple regression analysis	None/ Academic motivation (AMS)	0	+	+	0/-	+
McCredie & Kurtz (2020) [43]	Undergraduate students (n = 143), US	Raw correlation/Partial correlation	None	0	0	+	0	0
Noftle & Robins (2007) [44]								
sample 1	Undergraduate students in psychology (n = 10,497), US	Correlation analysis/Regression analysis	None/Gender, SAT verbal, SAT math	-	+/0	+	-	+/0
sample 2	Undergraduate students (n = 475), US	Correlation analysis/Regression analysis	None/Gender, SAT verbal, SAT math	0	0	+	0	+/0
Paunonen & Ashton (2001) [45]	Undergraduate students in psychology (n = 717), CA	Partial correlation	Gender			+		0
Paunonen & Ashton (2013) [46]	Undergraduate students in psychology (n = 652), CA	Partial correlation	Gender	-		+		0
Smidt (2015) [47]								
sample 1	College students (n = 465), DE	Correlation analysis/Multiple linear regression analysis	None/Gender, age, immigration background, socio-economic status, school-leaving GPA	0	0	+	+	0
sample 2	University students (n = 238), DE	Correlation analysis/Multiple linear regression analysis	None/Gender, age, immigration background, socio-economic status, school-leaving GPA	0/-	0/-	+	0	0/+
Vedel, Thomsen & Larsen (2015) [48]	University students (n = 1,067), DK	Correlation analysis/Multiple regression analysis	None	0	+/0	+	0	+

Studies reported in Table 1 show that conscientiousness is the most meaningful dimension of personality for academic performance. This evidence is in line with other results reported in the literature on adult outcomes such as productivity, earnings, work attendance and health-related behaviours [8, 49–51]. Borghans et al. [1] define conscientiousness “the degree to which a person is willing to comply with conventional rules, norms, and standards”, where the proactive side of the need for achievement and commitment to work combines with the inhibitive side of moral scrupulousness and cautiousness [52]. Burks et al. [32] observe that the proactive (but not the inhibitive) aspects of conscientiousness are positive predictors of final college GPA. Psychological research suggests that achievement-oriented and self-disciplined students with a persistent, hard-working aptitude despite any sources of distraction obtain higher college grades [39, 52]. Additional empirical evidence indicates that the association between conscientiousness and students’ grades may derive from the mediation role of having a stronger achievement motivation, a dispositional propensity toward the academic effort, a positive self-perception of the own academic ability and more productive study habits [44, 53, 54].

The relationship between the Big Five personality traits other than conscientiousness and grades appears more heterogeneous and smaller in magnitude, with openness to experience the most sizeable factor [16].

Openness to experience is “the degree to which a person needs intellectual stimulation, change, and variety” [1]. Some studies have failed to identify significant associations while others have provided supporting evidence that openness to experience positively affects grades and GPA [37, 39, 40]. Since openness to experience relates positively to a deep interest in learning and intellectual curiosity, students who score higher on this factor may more easily study for a desire of knowledge, producing in this way positive effects on their academic performance [36, 55].

The extant literature has produced little evidence that extraversion, agreeableness and emotional stability significantly correlate to grades. Extraversion describes the interpersonal qualities of being outgoing, sociable and talkative, and a general propensity to experience positive emotions [56]. Some studies have observed a negative relationship between extraversion and performance at college [33, 38, 44]. A possible explanation for these findings is that the need for social interactions of extroverted students negatively affects their ability to accomplish hard tasks. On the other hand, Komarraju et al. [42] find a positive correlation between extraversion and extrinsic motivation among US undergraduate students and they attribute it to the fact that, in pursuing academic goals, extroverted students aim to satisfy their need for achievement more than being interested in the intrinsic rewards of learning.

Meta-analyses of agreeableness and post-secondary performance indicate weak evidence of a positive relationship [57–59]. For instance, Farsides and Woodfield [37] argue that lower rates of absences from seminars [53] may mediate the positive correlation between agreeableness and final grades among US college students. Since, by definition, agreeable individuals have an altruistic, compliant and tender-minded nature [56], Komarraju et al. [42] attribute the positive performance of the more agreeable students to a higher degree of motivation and the consequent propensity towards engagement and cooperative behaviour in the classroom.

The empirical literature that informs the relationship between emotional stability and post-secondary performance has produced contradictory results. In most cases, the evidence does not show statistically significant correlations. While some studies report a positive association with academic performance [33, 34, 40], other studies present findings that move to the opposite direction [42, 44, 60], and meta-analytic reviews of the psychological literature suggest that emotional stability is not a strong predictor of performance [57, 59]. Emotionally stable individuals are less likely to experience feelings of anxiety, internal distress and self-consciousness than neurotic individuals [56]. With respect to the academic performance, on the one hand, emotionally stable students are expected to be more productive than neurotic students because of their better ability to manage academic stress [61]. On the other hand, emotionally stable students may make less effort to prepare their exams because they are conscious of being able to adapt more readily to adverse events or, equivalently, face a lower expected cost from a bad outcome. In support of this explanation, Delaney et al. [53] observe that students who score higher on neuroticism are those more likely to do extra hours of study.

## Data

Data sources are a web-based survey and administrative data from a cohort of freshmen at the University of Messina, a large public university located in southern Italy, which enrolled, according to public records, over 23,000 students in any degree programme (*course of study*) for the academic year 2016–2017.

The Italian university system is part of the European Higher Education Area (EHEA), a collaboration across countries which promotes the integration of higher education systems and the mutual recognition of academic qualifications. Undergraduate education is organized in three-year study programmes corresponding to a bachelor’s degree (*Laurea*), while a second level of academic qualification is obtained after two years of postgraduate studies corresponding to a master’s degree (*Laurea magistrale*). In addition, a limited number of study programmes for the access to regulated professions in Italy, i.e. Architecture, Dentistry, Human Medicine, Law, Pharmacy and Veterinary, is structured in a unique cycle lasting either five or six years, after which students hold a master’s degree (*Laurea magistrale a ciclo unico*).

We conduct our research on undergraduate students of either first-cycle or single-cycle degree courses aged between 18–24 years at the final deadline for matriculation by using the 90^th^ percentile of the age distribution as reference threshold to fix the range. There are several reasons to examine the academic performance of freshmen students. First of all, the first year of study is a critical step in the educational pathway, as suggested by the fact that university dropouts mostly occur during this time. In Italy, the percentage of freshmen students of the cohort 2016–2017 who abandoned university after the first year was around 12 per cent for first-cycle degree courses and 7.5 per cent for single-cycle degree courses [62]. Moreover, the ability with which a student copes with a different and more independent learning system in the first year enhances the ability to manage the workload in the subsequent years and, therefore, the likelihood that the student will persist in education. Finally, the first-cycle and single-cycle degree programmes usually provide more common and basic courses in the first year and more differentiated and specialized courses in the subsequent years.

We obtained anonymised data from the Information System Office of the University of Messina for use at the aggregate level and research purposes. All data used for this study derive from personal information required in the matriculation form that subjects submitted as general prerequisites for matriculation and management of student career. Written and informed consent was obtained from students during the matriculation process. The questionnaire, although not approved by an institutional review board, constitutes a macroscopic investigation that the University of Messina yearly conducts on the entire student population for improving teaching quality according to established procedures and with the consent of the university—collegial and administrative- bodies. While administrative data provides us with a wide range of student’s personal and schooling information, the questionnaire allows us to collect detailed information on parental characteristics–educational attainment, occupational status and industry–aside from on the student’s personality profile.

### Personality traits

The personality questionnaire administered to the students is the Ten-Item Personality Inventory (TIPI) with the adaptation for Italian subjects by Chiorri et al. [27] of the commonly adopted questionnaire of Gosling et al. [28]. The TIPI is a short-form questionnaire based on a set of paired items, each one containing two descriptors that capture the poles of the broad dimensions of personality. Respondents rate to what extent each item is self-descriptive on a seven-point Likert-type scale. We obtain the measures of the Big Five traits by first recoding the reverse-scored items and then averaging the scores of the two items for each scale. A detailed discussion on the potential limitations and advantages of using the TIPI for economic research based on survey data is provided in S1 Appendix.

### The measure of academic performance

The evaluation of volume learning in the context of the EHEA is based on the European Credit Transfer System (ECTS), a tool that allows to compare the workload associated with the courses and to transfer academic qualifications from a higher education institution into another. In Italian universities, one ECTS credit (known as university credit) is conventionally equivalent to 25 hours of work, including lessons, exercises and individual study. To pass the exams students must obtain grades ranging from 18/30 (the minimum score) to 30/30 and, in case of failure, the test score is not reported in the university transcript records. Educational achievement is measured as the grade point average (GPA), that is the average of the grades on the completed courses, weighted by the number of university credits assigned to each course. Further discussion about the main characteristics of the GPA is available in the S1 Appendix.

To account for possible sources of heterogeneity in the characteristics of the study programmes, such as class scheduling, teaching methods and performance evaluation, affecting the distribution of student scores and hence their comparability, we standardize the GPA within each course of study. To address further distributional issues, we also considered an alternative measure of university performance obtained from the percentile ranks of the GPA scores by course of study. Since the estimated effects are not qualitatively different from the analyses using the standardized values of GPA, we employ for simplicity this latter measure in the empirical analysis. A replication of the main results using the percentile ranks of GPA is included in S1 Table 1 in S1 Appendix.

### Descriptive statistics

The dataset consists of 3,242 students after excluding 75 observations for which information about school background is not available or incomplete and 860 observations on students not reporting grades.

**Fig 1:**
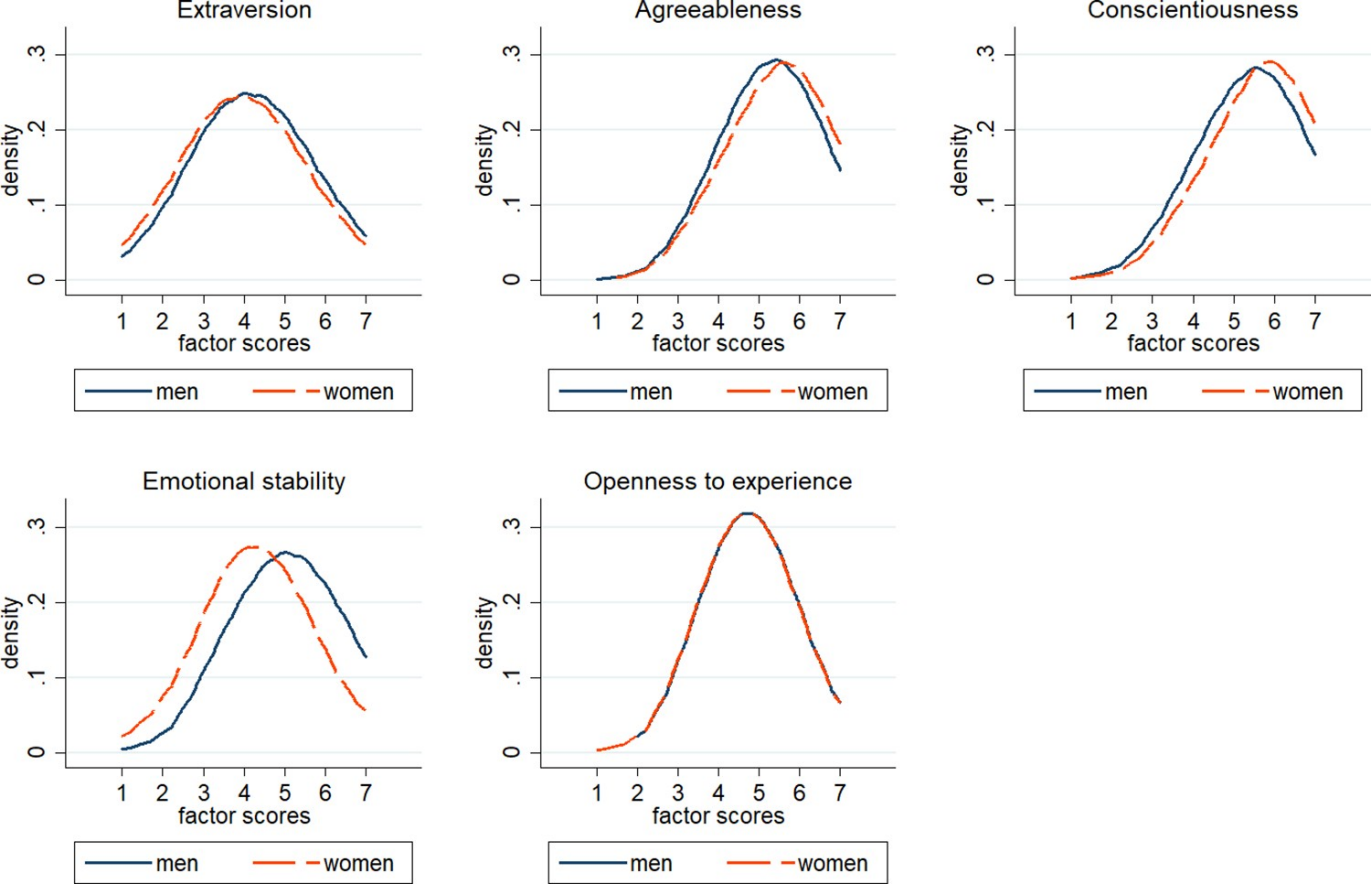
Density distribution of the Big Five trait scores by gender.

Table 2 reports the descriptive statistics of the variables considered in the econometric analysis. Table 3 presents the descriptive statistics of the Big Five trait measures, broken down by gender. Using a set of nonparametric Wilcoxon rank-sum tests, the null hypothesis that the personality scales are equal in distribution between the samples of women and men is rejected for all the domains (*p-value* = .000) except for openness to experience (*p-value* = .689). The Big Five density distribution by gender is also graphically presented in Fig 1. With respect to men, women score higher on agreeableness and conscientiousness but lower on extraversion and emotional stability. However, the effect sizes (Cohen’s *d*) for such scales are low (mean absolute value *d* = .166) except for emotional stability (*d* = 0.602). The latter result is consistent with the findings from psychological literature that women are more inclined to experience anxiety and internal distress than men since the early stages of adolescence [63]. The results are available in S1 Table 2 in S1 Appendix.

**Table 2:** Descriptive statistics.

	(2)	(3)	(4)	(5)
Variables	Mean	Standard deviation	Min.	Max.
Grade point average (GPA)	25.070	2.614	18	30
Extraversion	4.074	1.354	1	7
Agreeableness	5.406	1.055	1	7
Conscientiousness	5.534	1.116	1	7
Emotional stability	4.633	1.252	1	7
Openness to experience	4.766	.940	1	7
Female	.590	.492	0	1
Age at 30 Dec 2016	19.975	1.171	17.966	24
*Type of upper secondary school*				
*Liceo* for classical studies	.179	.384	0	1
*Liceo* for scientific studies	.398	.490	0	1
*Liceo* for other studies	.183	.387	0	1
Technical/vocational school	.240	.427	0	1
*Educational attainment*				
*Father*				
up to lower secondary school degree	.339	.473	0	1
upper secondary school degree	.451	.498	0	1
graduate in matched field	.054	.225	0	1
graduate not in matched field	.156	.363	0	1
*Mother*				
up to lower secondary school degree	.299	.458	0	1
upper secondary school degree	.481	.500	0	1
graduate in matched field	.044	.206	0	1
graduate not in matched field	.176	.381	0	1
*Occupation*				
*Father*				
unemployed, in education	.094	.292	0	1
employee	.594	.491	0	1
self employed	.243	.429	0	1
other	.014	.117	0	1
retired	.055	.228	0	1
*Mother*				
unemployed, in education	.296	.456	0	1
employee	.523	.500	0	1
self employed	.084	.278	0	1
other	.086	.281	0	1
retired	.011	.103	0	1
*Industry*				
*Father*				
business/personal services, public administration	.326	.469	0	1
professional, scientific, technical activities	.118	.323	0	1
manufacturing, construction	.064	.246	0	1
other	.491	.500	0	1
*Mother*				
business/personal services, public administration	.238	.426	0	1
professional, scientific, technical activities	.040	.197	0	1
manufacturing, construction	.011	.103	0	1
other	.711	.454	0	1
*ERC sectors*				
Social Sciences and Humanities	.447	.497	0	1
Physical Sciences and Engineering	.085	.279	0	1
Life Sciences	.468	.499	0	1
*Municipality / province of residence*				
City of Messina	.278	.448	0	1
Other city in the province of Messina	.306	.461	0	1
Other city in the Sicilian region	.183	.387	0	1
City of Reggio Calabria	.100	.300	0	1
Other city in the province of Reggio Calabria	.094	.292	0	1
Other city in the Calabria region	.031	.175	0	1
City in another region	.007	.084	0	1

*Note*. The grade point average (GPA) variable is measured as the average of the grades obtained in the first year of the study programme weighted by the credits associated with those grades. The variable “graduate in matched field” is a dummy variable on whether father/mother graduated in a field of study analogous to the child.

**Table 3:** Descriptive statistics of the Big Five personality traits by gender.

	Men				Women			
	(N = 1,330)				(N = 1,912)			
	Mean	Standard deviation	Min.	Max.	Mean	Standard deviation	Min.	Max.
Extraversion	4.202	1.323	1	7	3.985	1.369	1	7
Agreeableness	5.298	1.037	1	7	5.480	1.060	1.5	7
Conscientiousness	5.384	1.119	1	7	5.639	1.103	1	7
Emotional stability	5.061	1.170	1	7	4.334	1.222	1	7
Openness to experience	4.775	0.939	2	7	4.759	0.941	1	7

## Empirical strategy

Consider the following equation describing the main determinants of student performance, measured with the grade point average (GPA), for student *i =* 1, 2,…, n at time t+1 (first year of the degree programme):

$$GPA_{i\;(t+1)}=\alpha +\sum_{j=1}^{5}\beta_{j}{\mathrm{factor}}_{i,j(t)}+\gamma \;X_{i}+\delta \;Z_{i}+\varepsilon_{i},$$ (1)

where *factor* represents the Big Five personality trait *j =* 1, 2, …, 5 at time *t* (matriculation time); *X* is a vector of predetermined characteristics, *Z* is a vector of additional controls and *ε* is an unobserved error term. The coefficient of interest, *β*_*j*_, estimates the average effect of a one-point increase in the personality trait *j* on the GPA. The different levels of personality traits are a function of individual and family-level characteristics that could influence academic performance. A potential problem of omitted variable bias arises from ignoring the quantitative significance of these channels, so that the estimated effects of personality traits on GPA will be biased due to the correlation between the personality trait variables and the unobserved error term containing the omitted factors. The identification strategy relies on the conditional independence assumption to solve the selection problem by including a range of potentially confounding factors as control variables in the regression, so that the covariance between the personality trait variables and the error term is equal to zero once conditioning on the set of predetermined characteristics *X*; or, in other words, the levels of personality traits are as good as randomly distributed across students. Specifically, we consider gender, age, school background and parents’ information as potentially explanatory factors of academic performance that might relate to personality traits. Therefore, changes in the magnitude of the coefficients on personality traits after the inclusion of predetermined characteristics in the model indicate that personality traits are partly capturing the influence of these variables on GPA.

### Individual demographics

According to psychological studies about the correlation between personality traits and gender, women are more agreeable and less emotionally stable, on average, than men [52, 64, 65]. In addition, previous literature focusing on the gender gap in developed countries suggests that females tend to outperform males in school grades and that a male disadvantage emerges in grade point average at the stage of post-secondary education [66–68]. Age is associated with the maturation of personality traits over the life cycle, with the most noticeable changes occurring in the stages of childhood and adolescence [69–72]. On the one hand, a strand of literature reported negative effects of being younger on academic performance since the primary school [73, 74]. On the other hand, further evidence observed that younger students obtained better academic outcomes than their older counterparts at the undergraduate level [75, 76].

### School background

The acquisition of human capital from attending school is expected to promote student success in tertiary education [25]. The school background variables consist of indicators for categories of upper secondary school. The Italian upper secondary school education typically lasts five years (from age 14 to 19) and concludes with the State Examination—a set of national exams whose subjects depend on the type of school attended. The school leaving qualification or *diploma di maturità* enables the student to access higher education and provides a final score reflecting the general background knowledge acquired through schooling years. Since noncognitive skills play a role in the human capital accumulation process and contribute to explain scholastic success [1, 77, 78], the educational achievement captured by the measure of *diploma* score also depends on personality factors. Accordingly, including a covariate in the model which is endogenous respect to the Big Five traits would not allow to correctly identify the effects of such regressors of interest on university performance, as shown in Angrist and Pischke [79]. However, since individuals at the point of upper secondary school entry were hardly able to forecast their personality formation during adolescence, it is safe to assume that the Big Five trait scores measured at the age of university entry did not retrospectively influence the type of school choice. The indicators for upper secondary school categories allow us to distinguish *licei* providing more theoretical and university-oriented skills from technical and vocational schools providing professional training for direct access to the job market. School categories are useful to gather information on the starting human capital endowment of students in the context of the Italian education system, where required effort and study subjects may vary markedly across school programmes. Notice also that prospective students are free to choose any undergraduate programme regardless of the type of school attended.

### Parents’ information

The parental variables are indicators of educational level, occupational status and industry. Parental investments in noncognitive skills during the childhood support the development of children’s positive qualities that favour knowledge acquisition and hence student performance [14, 80]. Additional evidence derives from empirical studies testing the positive effects of parental education and socio-economic conditions on the academic performance of undergraduate students [81–85]. If this is the case, omitting such information would not allow to disentangle the parental contribution to GPA from the effects of the personality trait scores. Personality traits may help also predicting student’s autonomy in the choice of the field of study with respect to parental pressures. We check for this hypothesis by including in some specifications a dummy on whether the parent holds a university degree or equivalent qualification related to the field of study of the child.

The vector of additional controls, *Z*, firstly includes the student’s field of study. Following the classification used by the European Research Council (ERC) to organize disciplines with coherent research domains, we group students in three fields of study or ERC sectors: Social Sciences and Humanities, Physical Sciences and Engineering, and Life Sciences. Although the enrolment decision is a selection process reflecting students’ preferences for the degree programmes, we show that the distribution of personality traits across majors of study, holding the conditional independence assumption, is as good as random once controlling for the set of predetermined characteristics *X*. Moreover, vector *Z* accounts for individual-specific municipality/province of residence fixed effects that may help explaining heterogeneity in the outcome variable for the following reasons. First, the time for travelling to and from the university may differ significantly across students of different areas and affect their performance. Second, the short-term opportunity-cost of studying versus leisure time may vary significantly across different (although close) areas. Third, different expectations on the job opportunities in the local labour market may be another source of variation in academic performance. Following the same logic as above, since the number of university credits is a function of personality traits, we cannot include this information in the set of controls [79].

Since students of the same degree programme are exposed to common unobservable components that influence their university pathway and involving, for instance, interactions with the peers, class size, course content and teaching style, we account for correlated observations within a degree programme by adjusting standard errors for clustering.

As mentioned in the previous section, the dataset does not include 860 observations on students not reporting grades. While the fact that a sizeable proportion of students do not register any exams during the year is quite common in public and large Italian universities, the estimates could be biased if the selection process depends on unobservable factors which influence the GPA and relate to personality traits. However, the Big Five traits appear balanced between the groups of students with and without grades, and the estimates are robust to fitting a double hurdle model which accounts for left censoring in the GPA scores.

We consider the validity of the empirical approach used in this paper against potential endogeneity concerns arising from measurement error, reverse causality and omitted variables.

First, an important issue is the influence on the estimates of measurement error possibly contained in self-reported factor scores. For instance, even unconsciously respondents may distort self-reports of personality traits to respond in a socially desirable manner [16]. However, several facts reassure us on this respect. First, the applicants who participated in the web-survey were aware that the data provided by filling in the personality inventory served research purposes only. Second, if the measurement error is classical (random), the only effect on estimates is on standard errors that, becoming larger, determine a reduction in the statistical significance of estimated coefficients. Third, the literature suggests how induced faking behaviour in a hypothetical scenario at the time of university admission does not affect the criterion validity of personality domain-scores relative to examination performance [86] and the impact of social desirability on the relationship between the Big Five personality traits and cumulative college GPA is minimal [87].

Second, a possible objection against the causal interpretation of the estimates is that dealing with the exams could have an impact on personality traits. In that case, the estimates in the regression model would be biased due to reverse causality–i.e., the outcome variable would affect personality traits in the opposite direction. However, personality traits were measured when students enrolled in their first year of undergraduate courses, that is in a time when they did not still have any information regarding the exams. In addition, the evidence in previous literature suggests that dramatic shifts in personality are unlikely to happen in young adulthood and, therefore, over the short-length period considered [88, 89].

Third, the strategy we employ in this paper assumes that the rich set of observed characteristics included in the model allows to control for the main sources of endogeneity in personality traits (selection on observables). Looking at the movements of the coefficients of interest, if the inclusion of additional control variables does not affect the magnitude of the point estimates the evidence can be considered as supporting the causal interpretation of the relationship. However, we evaluate the possibility that the error term still captures unobservable factors that influence the estimated results (selection on unobservables). Following Bellows and Miguel [29] and Oster [30], we test the robustness of our results by assessing to what extent the selection on unobservables should be relevant respect to the selection on observables to explain the entire effects of personality traits.

The data analysis reported in this study was performed with STATA software, version 13.0 (Stata Corporation, College Station, TX, USA). The threshold for statistical significance (*alpha level*) is *p* < .05. To facilitate the interpretation of the results, we standardize the personality trait scores and the continuous independent variables to have zero mean and standard deviation of one.

## Results

Table 4 reports the estimation results of the effects of the Big Five personality traits on GPA. The stability of the estimated parameters on the Big Five personality traits is evaluated considering a number of specifications with additional sets of controls.

**Table 4:** Estimated effects of the Big Five personality traits on GPA.

	(1)	(2)	(3)	(4)
Extraversion	-0.025	-0.025	-0.024	-0.022
	(0.019)	(0.018)	(0.019)	(0.018)
Agreeableness	-0.037	-0.039	-0.041	-0.037
	(0.023)	(0.023)	(0.023)	(0.024)
Conscientiousness	0.110***	0.094***	0.093***	0.092***
	(0.019)	(0.017)	(0.017)	(0.017)
Emotional stability	-0.057*	-0.035	-0.031	-0.030
	(0.027)	(0.024)	(0.023)	(0.023)
Openness to experience	0.036*	0.039*	0.038*	0.038*
	(0.018)	(0.018)	(0.017)	(0.017)
Female		0.140**	0.138**	0.148**
		(0.044)	(0.046)	(0.043)
Age		-1.049*	-1.114*	-0.978
		(0.494)	(0.493)	(0.500)
Age squared		1.021*	1.089*	0.957
		(0.499)	(0.497)	(0.505)
*Type of upper secondary school*				
*Liceo* for scientific studies		-0.094	-0.101	-0.103
		(0.056)	(0.055)	(0.053)
*Liceo* for other studies		-0.168***	-0.173***	-0.164***
		(0.039)	(0.036)	(0.039)
Technical/vocational school		-0.347***	-0.345***	-0.353***
		(0.064)	(0.059)	(0.057)
*ERC sectors*				
Physical Sciences and Engineering		0.093	0.077	0.060
		(0.047)	(0.049)	(0.049)
Life Sciences		0.055	0.055	0.065
		(0.050)	(0.054)	(0.054)
Parental controls			YES	YES
Municipality/ province of provenience				YES
Constant	-0.000	0.035	0.198	0.232*
	(0.005)	(0.056)	(0.101)	(0.098)
Observations	3242	3242	3242	3242
*R* ^2^	0.014	0.039	0.049	0.056
F	7.251	14.940	81.575	105.332
*p*-value	0.000	0.000	0.000	0.000

*Note*. The omitted category of upper secondary school is the *liceo* for classical studies. The omitted category of ERC sector is Social Sciences and Humanities. Parental controls include educational attainment, occupational status and industry. Significance level (*: *p* < .05, **: *p* < .01, ***: *p* < .001) based on robust standard errors (reported in parenthesis), clustered at the course of study level (46 clusters).

The findings suggest that the coefficients on the Big Five factor scores are substantially robust to the inclusion of the control variables. Conscientiousness and openness to experience exhibit positive and statistically significant coefficients, while the other Big Five traits do not appear to have a significant impact on GPA. Conscientiousness has the more intensive effect on GPA, which is consistent with the literature emphasizing the role played by motivational aspects and self-discipline on student achievement. Specifically, one standard deviation increase in conscientiousness is associated with an increase of 9.3 percent of a standard deviation in GPA. One standard deviation increase in openness to experience raises 3.8 percent of a standard deviation GPA. This result is in line with the view that the positive disposition toward intellectual engagement and novel ideas of the open to experience individuals determines a more intense interest in knowledge acquisition and, predictably, more positive achievement scores [36, 55].

The choice between *liceo* and technical or vocational school is associated with path differences in university performance. As predictable, students who received a more theoretical secondary education appear to outperform those with a technical or vocational background. In addition, students who attended other categories of *licei* that are more focused on visual arts, architecture and design, foreign languages, music or human sciences with respect to humanities or scientific disciplines perform less well than students who attended the *liceo* for classical studies.

According to a literature highlighting the existence of gender differences in academic performance, the results suggest that women obtain higher GPA scores than men. As discussed in Section 3, gender differences emerge in the density distribution of the Big Five trait scores, the only exception being openness to experience. However, the results from regression analyses including interaction terms between the Big Five traits and the gender dummy in the specifications suggest that the estimated effects are not heterogeneous between men and women (S2 Table 1 in S2 Appendix).

In addition to the observed controls included in the main analysis, further information on the family socioeconomic conditions, as well as the number of members, is available for a subsample (2341 of 3242, equal to 72 percent) of students who submitted the Equivalent Economic Situation Indicator (*ISEE*) certification to obtain a reduction in the tuition fees or other social benefits. When these controls are included in the regression analysis, the estimated effects remain qualitatively unchanged with respect to the main results (S2 Table 2 in S2 Appendix).

We conduct several exercises to verify the robustness of our results: (i) we replicate the analysis dropping out the observations reporting extreme values on agreeableness and conscientiousness to take into account the negatively skewed distributions of these traits for the estimates; (ii) we consider potential patterns of selection in the group of students who did not register any exam respect to students with grades by showing that personality traits are balanced between these categories and that the results of a double hurdle regression analysis modelling the zero values for GPA is consistent with the main results in this section; (iii) we demonstrate that personality traits can be considered as randomly distributed across fields of study once conditioning on the set of predetermined characteristics; (iv) we test the robustness of the estimates to selection on unobservables using the methodologies proposed by Bellows and Miguel [29] and Oster [30]. All these robustness checks are presented in the S2 Appendix.

## Concluding comments and policy implications

This paper provides insights that the Big Five dimensions of personality play a role in explaining the documented heterogeneity in student achievement and adds a piece of evidence to a strand of literature that attempts to trace the fundamentals of the relationship between personality traits and post-secondary performance. To address this research issue, we have taken advantage of an ample set of information about a cohort of freshmen students within the degree programs of a large public university in Italy. Additional specification checks verify that the estimated effects of personality traits are robust to endogenous factors informing the selection rule of the degree programmes. Our findings suggest that conscientiousness and openness to experience are positive personality traits in determining the grade point average of the exams that students passed throughout their first university year. In particular, the observational estimates reinforce the explanation that the aptitudes for dutifulness and self-discipline help conscientious individuals to sustain the enduring commitment to study required to achieve better performance outcomes. In addition, openness to experience appears a strategic trait for knowledge acquisition, highlighting how intellectual engagement and the ability to develop an independent approach to study are critical qualities to build up professional expertise that is valuable in the labour market.

To investigate to what extent the estimated results can be interpreted as evidence of a causal relationship between the Big Five personality traits and academic performance, we have implemented the empirical techniques proposed by Bellows and Miguel [29] and Oster [30]. We have found that, while for conscientiousness selection on unobservables should be strictly greater than the unit relative to selection on the observed variables to attribute the entire treatment effect to selection bias, in the case of openness to experience the estimated effect is likely to be downward biased by selection on unobservables.

A related literature on this topic argued that narrow-level traits could predict more accurately academic performance than solely relying on the broad dimensions of personality [45, 46]. Suggesting the findings of this study that conscientiousness and, to a lower extent, openness to experience, are the most relevant factors in explaining student grades, future research should primarily focus on low-level facets of such personality traits.

Advances in the comprehension of the relationship between noncognitive skills and student achievement are fundamental to identify strategies improving the competitiveness of education systems, especially for countries as Italy that, for instance, show higher indices of university dropouts among developed economies.

The results of this research support public investments in intervention programmes with the objective of enhancing the Big Five personality traits that are positively associated with productivity in adulthood: educational strategies should be designed to foster learning skills and intellectual engagement between childhood and adolescence [90]. Intervention programmes in this area have already proved to be effective, showing that adequate mentoring and training practices–either in the classroom or with extracurricular and social activities–can produce persistent improvements in intellectual engagement and behavioural changes [20, 90–93]. These intervention programmes are therefore expected to have a significant impact on the treated subjects in terms of the future investments in higher education.

Second, university institutions may more effectively emphasize the resources of talented students who are highly motivated and hardworking (conscientiousness), intellectually curious and creative (openness to experience) by offering them degree programmes that embrace different academic disciplines. In this spirit, top universities such as Harvard University, Massachusetts Institute of Technology and University of Oxford have introduced multidisciplinary courses that integrate Humanities with Science and Engineering.

Finally, university institutions may consider that differences in individuals’ personality traits influence their approach to learning and, consequently, they may seek to support students through organizational changes and targeted services fostering better productivity levels. For instance, universities may consider introducing compulsory attendance rules and the opportunity for students to take multiple exams, each one in partial fulfilment of the knowledge requirements for the completion of the course, instead of taking a single overall exam. On the one hand, these settings may serve as commitment strategies for students who are less diligent and self-disciplined to prevent inefficient procrastination. On the other hand, students who lack intellectual curiosity may benefit from engaging in a series of short-term deadlines to maintain the study effort. Understanding which strategies can have a positive impact on the performance of undergraduate students with a personality profile that is less rewarding in the university system is an interesting pathway for future research.

## Supporting information

S1 Appendix. Data This Appendix includes further results discussed in Section 3 (“Data”). (DOCX)

S2 Appendix Results and robustness checks. This Appendix includes further results discussed in Section 5 (“Results”) and presents the robustness checks.
(DOCX)
