# Supplementary material for: The influence of personality traits on university performance: Evidence from Italian freshmen students
Source: PLoS One. 2021 Nov 3;16(11):e0258586. doi: 10.1371/journal.pone.0258586 (PMC8565773; doi:10.1371/journal.pone.0258586)
Supplement: S1 Appendix. Data — (DOCX) [file pone.0258586.s001.docx]

**S1 Appendix**

**The Ten-Item Personality Inventory (TIPI)**

The TIPI is a short-form questionnaire based on a set of paired items, each one containing two descriptors that capture the poles of the broad dimensions of personality. Using a brief psychological instrument, as documented in the seminal paper of Gosling et al. [1] and further studies introducing translated versions of the original questionnaire [2-7], implies that the TIPI presents, in comparison to longer multi-item scales, low levels of internal consistency, reflecting the degree of correlation among items related to the same underlying construct. Instead, the TIPI emphasizes content validity considerations about the extent to which the measures are representative of the broad domains of personality at the cost of relatively low inter-item correlations. Following this reasoning, the selection of the items has the objective to minimize redundancy among descriptors and preserve content coverage [1]. In our study, the Spearman’s correlation coefficients for the paired items of the Big Five scales are all statistically significant (*p-value*<.000) and follow the expected patterns (*ρ_extra_*=-0.387, *ρ_agree_*=-0.110, *ρ_consc_*=-0.402, *ρ_emostab_*=-0.287, *ρ_open_*=-0.092).

The factors scores are the averages of the paired items for each scale once recoding the reverse-scored items. The average is better instructive of the data than using the items *per se*, providing a central tendency for the representation of the breadth of each factor. Related to this, Gosling et al. [1] argue that the retest stability of the scales over time is better informative of the reliability of the instrument than internal consistency, which depends on a small number of items per scale suited to meet content validity. The average value of the test-retest correlations was .72 in the original TIPI [1] and .85 in the Italian translation by Chiorri et al. [2], the latter case ranging from .77 (extraversion) to .90 (conscientiousness). These studies present further evidence on the construct validity of the TIPI, evaluating the extent to which the instrument represents the conceptual structure of the Big Five by means of convergent and discriminant correlations. In the Italian-revised version, convergent correlations with the well-established 44-item Big Five Inventory (BFI, [8]), that indicate how close the TIPI scales are to variables measuring the same underlying constructs, were significantly higher than discriminant correlations ( i.e. the extent to which the scales diverge from other scales measuring conceptually unrelated constructs) whose highest absolute value was *r*=.35 [2]. In addition, the patterns of external correlates with other constructs (i.e., affect, self-esteem, optimism, emotional regulation, and social desirability) did not statistically differ from those of the BFI.

Although internal consistency is an issue of practical importance that may hinder the relationship between the factor scores and the outcome variable, there are good reasons to use a short-form psychological questionnaire. First, given its brevity, the TIPI is an appropriate instrument for assessing the Big Five constructs at the group level in the context of survey data, in which the presence of numerous questions and time costs do not allow the use of long scales [9] (see, for instance, Erol and Orth [10] for an application of the TIPI in the National Longitudinal Survey of Youth). Second, the TIPI eliminates item redundancy, which induces frustration and boredom on the respondents repeatedly facing the same question over multiple items [1]. Third, contrary to instruments involving several items per scale, subjects’ evaluations in brief questionnaires are less likely to be affected by anchoring effects, which occur when the value attributed to an item establishes an initial reference point to rate the following items.

**The grade point average (GPA)**

Educational achievement is measured as the grade point average (GPA), that is the average of the grades on the completed courses, weighted by the number of university credits assigned to each course. Reflecting a trade-off between quality (average grades) and quantity (university credits), to the extent that students who invest great effort in academic preparation are less productive in terms of number of courses completed during the year, a comprehensive measure of the student’s performance is not available, though the relevant literature considers GPA as the most convenient and accurate approximation. Ideally, one should rely on experiments or quasi-experiment techniques that address the non-random distribution of credits across students to analyse in detail the nature of the relationship between credits and student grades. Although we cannot directly test the extent to which students trade credits for average grades, the combination of total number of credits and GPA that emerges is similar among students. The Spearman's rank correlation coefficient between GPA and total amount of credits earned is positive and statistically significant (*rho*=.27, *p*<.001). This result suggests that the frequency of courses completed during the year tends to be higher for students who invested more resources on academic preparation and performance.

**S1 Table 1. Estimated effects of the Big Five personality traits on GPA: percentile ranks of GPA scores.**

|  | (1) | (2) | (3) | (4) |
| --- | --- | --- | --- | --- |
| Extraversion | -0.008 | -0.008 | -0.008 | -0.007 |
|  | (0.006) | (0.006) | (0.006) | (0.006) |
| Agreeableness | -0.012 | -0.013^*^ | -0.014^*^ | -0.012 |
|  | (0.006) | (0.006) | (0.006) | (0.006) |
| Conscientiousness | 0.032^***^ | 0.028^***^ | 0.027^***^ | 0.027^***^ |
|  | (0.006) | (0.005) | (0.005) | (0.005) |
| Emotional Stability | -0.017^*^ | -0.010 | -0.009 | -0.009 |
|  | (0.008) | (0.007) | (0.006) | (0.006) |
| Openness to experience | 0.011^*^ | 0.012^*^ | 0.011^*^ | 0.011^*^ |
|  | (0.005) | (0.005) | (0.005) | (0.005) |
| Female |  | 0.039^**^ | 0.038^**^ | 0.042^**^ |
|  |  | (0.013) | (0.014) | (0.013) |
| Age |  | -0.321^*^ | -0.344^*^ | -0.300^*^ |
|  |  | (0.144) | (0.142) | (0.146) |
| Age squared |  | 0.313^*^ | 0.337^*^ | 0.294 |
|  |  | (0.146) | (0.144) | (0.148) |
| *Type of upper secondary school* |  |  |  |  |
| *Liceo* for scientific studies |  | -0.036^*^ | -0.038^*^ | -0.039^*^ |
|  |  | (0.017) | (0.017) | (0.016) |
| *Liceo* for other studies |  | -0.056^***^ | -0.057^***^ | -0.055^***^ |
|  |  | (0.013) | (0.012) | (0.013) |
| Technical/vocational school |  | -0.109^***^ | -0.109^***^ | -0.111^***^ |
|  |  | (0.019) | (0.017) | (0.017) |
| *ERC sectors* |  |  |  |  |
| Physical Sciences and Engineering |  | 0.025 | 0.020 | 0.015 |
|  |  | (0.013) | (0.014) | (0.014) |
| Life Sciences |  | 0.018 | 0.018 | 0.022 |
|  |  | (0.015) | (0.016) | (0.017) |
| Parental controls |  |  | YES | YES |
| Municipality/ province of provenience |  |  |  | YES |
| Constant | 0.496^***^ | 0.514^***^ | 0.561^***^ | 0.574^***^ |
|  | (0.002) | (0.016) | (0.029) | (0.028) |
| Observations | 3242 | 3242 | 3242 | 3242 |
| *R*^2^ | 0.014 | 0.040 | 0.050 | 0.058 |
| F | 7.629 | 18.090 | 100.890 | 160.148 |
| *p*-value | 0.000 | 0.000 | 0.000 | 0.000 |

*Note*. The table shows the estimated effects of the Big Five traits on academic performance, which is obtained from the percentile ranks of GPA scores by course of study. The omitted category of upper secondary school is the *liceo* for classical studies. The omitted category of ERC sector is Social Sciences and Humanities. Parental controls include educational attainment, occupational status and industry. Personality traits and continuous variables are standardized to have mean 0 and standard deviation of 1. Significance level (*: *p*<.05, **: *p*<.01, ***: *p*<.001) based on robust standard errors (reported in parenthesis), clustered at the course of study level (46 clusters).

**Descriptive statistics**

**S Table 2. Wilcoxon rank-sum test and Cohen’s *d* statistics: personality and gender.**

|  | Wilcoxon-rank sum test |  | Cohen’s *d* | |
| --- | --- | --- | --- | --- |
|  | p-value |  | Effect size | CI (95%) |
| Extraversion | 0.000 |  | 0.138 | 0.091, 0.231 |
| Agreeableness | 0.000 |  | -0.136 | -0.244, -0.103 |
| Conscientiousness | 0.000 |  | -0.224 | -0.299, -0.159 |
| Emotional stability | 0.000 |  | 0.602 | 0.534, 0.677 |
| Openness to experience | 0.682 |  | -0.013 | -0.053, 0.087 |

*Note.* The first column reports p-values from a Wilcoxon rank-sum test of the null hypothesis that the two independent samples of men (*N*=1,330) and women (*N*=1,912) are from populations with equal distribution. The second column reports the effect sizes (Cohen’s *d*) for the average differences in the Big Five personality traits between the samples of men and women (with confidence intervals, CI, at .95 level). Notice that the indicator variable for the two samples is equal to one if the gender is female.

**References**

1. Gosling SD, Rentfrow PJ, Swann WB. A very brief measure of the Big-Five personality domains. J Res Pers. 2003;37: 504–528. doi:10.1016/S0092-6566(03)00046-1

2. Chiorri C, Bracco F, Piccinno T, Modafferi C, Battini V. Psychometric properties of a revised version of the Ten Item Personality Inventory. Eur J Psychol Assess. 2015;31: 109–119. doi:10.1027/1015-5759/a000215

3. Hofmans J, Kuppens P, Allik J. Is short in length short in content? An examination of the domain representation of the Ten Item Personality Inventory scales in Dutch language. Pers Individ Dif. 2008;45: 750–755. doi:10.1016/j.paid.2008.08.004

4. Muck PM, Hell B, Gosling SD. Construct validation of a short five-factor model instrument: a self-peer study on the German adaptation of the Ten-Item Personality Inventory (TIPI-G). Eur J Psychol Assess. 2007;23: 166–175. doi:10.1027/1015-5759.23.3.166

5. Renau Ruiz V, Oberst U, Gosling S, Rusiñol J, Chamarro Lusar A. Translation and validation of the Ten-Item-Personality Inventory into Spanish and Catalan. Aloma Rev Psicol ciències l’educació i l’esport Blanquerna. 2013;0: 85-97–97.

6. Romero E, Villar P, Gómez-Fraguela JA, López-Romero L. Measuring personality traits with ultra-short scales: a study of the Ten Item Personality Inventory (TIPI) in a Spanish sample. Pers Individ Dif. 2012;53: 289–293. doi:10.1016/j.paid.2012.03.035

7. Storme M, Tavani JL, Myszkowski N. Psychometric properties of the French Ten-Item Personality Inventory (TIPI). J Individ Differ. 2016;37: 81–87. doi:10.1027/1614-0001/a000204

8. John OP, Naumann LP, Soto CJ. Paradigm shift to the integrative Big Five Trait taxonomy: history, measurement, and conceptual issues. Handbook of personality: Theory and research. 2008. doi:10.1016/S0191-8869(97)81000-8

9. Rammstedt B, Beierlein C. Can’t we make it any shorter? The limits of personality assessment and ways to overcome them. J Individ Differ. 2014;35: 212–220. doi:10.1027/1614-0001/a000141

10. Erol RY, Orth U. Self-esteem development from age 14 to 30 years: a longitudinal study. J Pers Soc Psychol. 2011;101: 607–619. doi:10.1037/a0024299
