# Supplementary material for: The influence of personality traits on university performance: Evidence from Italian freshmen students
Source: PLoS One. 2021 Nov 3;16(11):e0258586. doi: 10.1371/journal.pone.0258586 (PMC8565773; doi:10.1371/journal.pone.0258586)
Supplement: S2 Appendix — This Appendix includes further results discussed in Section 5 (“Results”) and presents the robustness checks. (DOCX) [file pone.0258586.s002.docx]

S2 Appendix

**Personality traits and gender differences**

**S2 Table 1. Estimated effects of the Big Five personality traits on GPA: personality and gender.**

|  | (1) | (2) |
| --- | --- | --- |
| Extraversion | -0.034 | -0.031 |
|  | (0.034) | (0.034) |
| Agreeableness | -0.042 | -0.046 |
|  | (0.034) | (0.034) |
| Conscientiousness | 0.094^**^ | 0.094^**^ |
|  | (0.028) | (0.027) |
| Emotional stability | -0.077^*^ | -0.071^*^ |
|  | (0.031) | (0.031) |
| Openness to experience | 0.075^*^ | 0.073^*^ |
|  | (0.031) | (0.031) |
| Female | 0.130^**^ | 0.128^*^ |
|  | (0.047) | (0.049) |
| Extraversion × Female | 0.018 | 0.015 |
|  | (0.039) | (0.039) |
| Agreeableness × Female | 0.002 | 0.004 |
|  | (0.032) | (0.033) |
| Conscientiousness × Female | 0.004 | 0.002 |
|  | (0.034) | (0.033) |
| Emotional stability × Female | 0.067 | 0.063 |
|  | (0.034) | (0.034) |
| Openness to experience × Female | -0.059 | -0.057 |
|  | (0.048) | (0.048) |
| Age | -1.055^*^ | -1.118^*^ |
|  | (0.498) | (0.497) |
| Age squared | 1.028^*^ | 1.093^*^ |
|  | (0.503) | (0.501) |
| *Type of upper secondary school* |  |  |
| *Liceo* for scientific studies | -0.096 | -0.103 |
|  | (0.055) | (0.054) |
| *Liceo* for other studies | -0.170^***^ | -0.176^***^ |
|  | (0.039) | (0.036) |
| Technical/vocational school | -0.353^***^ | -0.352^***^ |
|  | (0.064) | (0.058) |
| *ERC sectors* |  |  |
| Physical Sciences and Engineering | 0.088 | 0.073 |
|  | (0.047) | (0.050) |
| Life Sciences | 0.054 | 0.054 |
|  | (0.051) | (0.055) |
| Parental controls |  | YES |
| Constant | 0.054 | 0.213^*^ |
|  | (0.061) | (0.103) |
| Observations | 3242 | 3242 |
| *R*^2^ | 0.041 | 0.051 |
| F | 17.662 | 91.808 |
| *p-*value | 0.000 | 0.000 |

*Note*. The table shows the interaction effects of the Big Five traits on GPA between women and men. The omitted category of upper secondary school is the *liceo* for classical studies. The omitted category of ERC sector is Social Sciences and Humanities. Parental controls include educational attainment, occupational status and industry. Significance level (*: *p*<.05, **: *p*<.01, ***: *p*<.001) based on robust standard errors (reported in parenthesis), clustered at the course of study level (46 clusters).

**The Equivalent Economic Situation Indicator (ISEE)**

The Equivalent Economic Situation Indicator (ISEE) is a certificate based on information about income, asset situation and household composition that gives access to scholarship programmes, reduction in the tuition fees and other student benefits. Some students with high socioeconomic status (ISEE>Eur 60,000) did not provide the ISEE certificate since they were required to pay the maximum amount of tuition fees in any case. Other students did not request their ISEE because were exempted from paying tuition fees (e.g. students with maximum school leaving qualification or students eligible for disability benefits). For a more in-depth analysis of the stability of the results to socio-economic condition, we test the following alternative specifications: (i) we include the ISEE variable (measured as the standardized logarithms of the ISEE values) and the number of household members as regressors, but excluding the parental controls; (ii) we include the ISEE variable, the number of household members and the parental controls simultaneously. The estimated effects (S2 Table 2) remain qualitatively unchanged with respect to those presented in Table 4 of the Results section.

**S2 Table 2. Estimated effects of the Big Five personality traits on GPA: Equivalent Economic Situation Indicator (ISEE).**

|  | (1) | (2) |
| --- | --- | --- |
| Extraversion | -0.023 | -0.021 |
|  | (0.017) | (0.018) |
| Agreeableness | -0.037 | -0.037 |
|  | (0.026) | (0.026) |
| Conscientiousness | 0.110^***^ | 0.106^***^ |
|  | (0.022) | (0.022) |
| Emotional stability | -0.028 | -0.027 |
|  | (0.023) | (0.022) |
| Openness to experience | 0.040^*^ | 0.037^*^ |
|  | (0.018) | (0.018) |
| Female | 0.108^*^ | 0.101^*^ |
|  | (0.045) | (0.046) |
| Age | -0.500 | -0.499 |
|  | (0.646) | (0.621) |
| Age squared | 0.486 | 0.485 |
|  | (0.654) | (0.629) |
| *Type of upper secondary school* |  |  |
| *Liceo* for scientific studies | -0.073 | -0.080 |
|  | (0.058) | (0.059) |
| *Liceo* for other studies | -0.165^**^ | -0.176^**^ |
|  | (0.052) | (0.050) |
| Technical/vocational school | -0.372^***^ | -0.383^***^ |
|  | (0.063) | (0.057) |
| *ERC sectors* |  |  |
| Physical Sciences and Engineering | 0.136^*^ | 0.130^*^ |
|  | (0.058) | (0.059) |
| Life Sciences | 0.095 | 0.090 |
|  | (0.050) | (0.052) |
| Logarithm of ISEE | -0.024 | -0.016 |
|  | (0.015) | (0.016) |
| Size of the household | 0.029 | 0.024 |
|  | (0.030) | (0.030) |
| Parental controls |  | YES |
| Municipality/ province of provenience | YES | YES |
| Constant | -0.023 | 0.063 |
|  | (0.122) | (0.146) |
| Observations | 2341 | 2341 |
| *R*^2^ | 0.051 | 0.058 |
| F | 12.096 | 287.377 |
| *p*-value | 0.000 | 0.000 |

*Note*. The table shows the estimated effects of the Big Five traits on GPA once including the ISEE variable (measured as the standardized logarithms of the ISEE values) and the number of household members as regressors. The omitted category of upper secondary school is the *liceo* for classical studies. The omitted category of ERC sector is Social Sciences and Humanities. Parental controls include educational attainment, occupational status and industry. Significance level (*: *p*<.05, **: *p*<.01, ***: *p*<.001) based on robust standard errors (reported in parenthesis), clustered at the course of study level (46 clusters).

**Distributional issues**

It is worth noting that agreeableness and conscientiousness exhibit negatively skewed distributions (*skew*_a_=-.379 and *skew*_c_=-.645) and high average values (*m_a_*=5.40 and *m_c_*= 5.534, see Table 2), meaning that a distortion toward extreme scores may understate the actual association of these traits and variables having more symmetrical distributions, with implications for the evaluation of their effects on the outcome variable. The percentage of students reporting the maximum score either on agreeableness or conscientiousness (or on both scales) in the dataset is around 20.76 per cent. Specifically, 337 observations exhibit the maximum score on agreeableness and 497 on conscientiousness. Instead, very few students describe themselves with the lowest score on these traits (they are only one for agreeableness and five for conscientiousness). As a robustness check, we replicate the regression analysis dropping out the observations with extreme values on agreeableness and conscientiousness. The results are qualitatively similar to those obtained using the entire sample and are reported in S2 Table 3.

**S2 Table 3. Estimated effects of the Big Five personality traits on GPA: excluding the extreme values on agreeableness and conscientiousness.**

|  | (1) | (2) | (3) | (4) |
| --- | --- | --- | --- | --- |
| Extraversion | -0.019 | -0.018 | -0.017 | -0.015 |
|  | (0.023) | (0.023) | (0.023) | (0.022) |
| Agreeableness | -0.029 | -0.030 | -0.032 | -0.029 |
|  | (0.023) | (0.023) | (0.023) | (0.024) |
| Conscientiousness | 0.102^***^ | 0.091^***^ | 0.088^***^ | 0.086^***^ |
|  | (0.018) | (0.017) | (0.018) | (0.019) |
| Emotional stability | -0.061^*^ | -0.043 | -0.039 | -0.038 |
|  | (0.026) | (0.024) | (0.023) | (0.023) |
| Openness to experience | 0.048^**^ | 0.050^**^ | 0.048^**^ | 0.046^**^ |
|  | (0.017) | (0.016) | (0.016) | (0.017) |
| Female |  | 0.125^**^ | 0.118^**^ | 0.137^**^ |
|  |  | (0.044) | (0.044) | (0.041) |
| Age |  | -1.182^*^ | -1.247^*^ | -1.121^*^ |
|  |  | (0.498) | (0.522) | (0.526) |
| Age squared |  | 1.167^*^ | 1.236^*^ | 1.116^*^ |
|  |  | (0.496) | (0.520) | (0.524) |
| *Type of upper secondary school* |  |  |  |  |
| *Liceo* for scientific studies |  | -0.111 | -0.120 | -0.124 |
|  |  | (0.070) | (0.072) | (0.069) |
| *Liceo* for other studies |  | -0.224^***^ | -0.232^***^ | -0.225^***^ |
|  |  | (0.057) | (0.058) | (0.058) |
| Technical/vocational school |  | -0.361^***^ | -0.364^***^ | -0.374^***^ |
|  |  | (0.072) | (0.070) | (0.065) |
| *ERC sectors* |  |  |  |  |
| Physical Sciences and Engineering |  | 0.089^*^ | 0.075 | 0.060 |
|  |  | (0.041) | (0.045) | (0.045) |
| Life Sciences |  | 0.029 | 0.029 | 0.047 |
|  |  | (0.058) | (0.061) | (0.062) |
| Parental controls |  |  | YES | YES |
| Municipality/ province of provenience |  |  |  | YES |
| Constant | -0.009 | 0.072 | 0.282^**^ | 0.333^**^ |
|  | (0.009) | (0.055) | (0.097) | (0.105) |
| Observations | 2563 | 2563 | 2563 | 2563 |
| *R*^2^ | 0.014 | 0.039 | 0.051 | 0.059 |
| F | 8.928 | 11.306 | 43.546 | 141.058 |

*Note*. The omitted category of upper secondary school is the *liceo* for classical studies. The omitted category of ERC sector is Social Sciences and Humanities. Parental controls include educational attainment, occupational status and industry. Significance level (*: *p*<.05, **: *p*<.01, ***: *p*<.001) based on robust standard errors (reported in parenthesis), clustered at the course of study level (46 clusters).

**Students without grades**

The dataset does not include 860 observations on students not reporting grades. While the fact that a sizeable proportion of students do not register any exams during the year is quite common in public and large Italian universities, the estimates could be biased if the selection process depends on unobservable factors which influence the GPA and relate to personality traits.

However, the Big Five traits are balanced across students who earned university credits during the year and students without credits registered based on the comparison of the means (standardized differences) and variances (variance ratios). Following Normand et al. (2001) [1], standardized differences greater than .10 in absolute value would reflect unbalance between student groups, while the highest value we observe (the distance between group means for conscientiousness) is only .077. The variance ratios range between a minimum value of .916 (for agreeableness) and a maximum value of 1.06 (for extraversion), that are close to the recommended value of 1.0 and boundaries 0.5-2.0 [2]. Therefore, students do not seem to select depending on their endowment in terms of personality traits. The results are reported in S2 Table 4.

Although understanding the reasons explaining the rather high rate of students without grades (860 out of 4,102) is outside the focus of this paper, we attempt two related explanations that are i) little interest in the subjects of the selected course of study and ii) inadequate background and skills for coping with the effort required by a university study programme. The fact that this group reports, on average, a lower score on the school leaving certificate than the group of students with graded exams (*d=.33*) and a higher proportion of students with a technical or vocational school background (*d=-.12*) seems to corroborate this conjecture.

**S2 Table 4. Balance between the group of freshmen who earned credits in the academic year 2016-2017 and the group without credits.**

|  | Students with credits  (*N=3,242*) | | | Students without credits  (*N=860*) | | |  |  |
| --- | --- | --- | --- | --- | --- | --- | --- | --- |
|  | Mean | Variance | Skewness | Mean | Variance | Skewness | Std-diff | Var-ratio |
| Extraversion | 4.074 | 1.833 | 0.026 | 4.113 | 1.737 | 0.076 | -0.029 | 1.056 |
| Agreeableness | 5.406 | 1.112 | -0.379 | 5.341 | 1.214 | -0.491 | 0.060 | 0.916 |
| Conscientiousness | 5.534 | 1.246 | -0.645 | 5.448 | 1.241 | -0.423 | 0.077 | 1.004 |
| Emotional stability | 4.633 | 1.569 | -0.072 | 4.607 | 1.611 | -0.126 | 0.020 | 0.973 |
| Openness to experience | 4.766 | 0.884 | 0.076 | 4.799 | 0.922 | 0.195 | -0.035 | 0.959 |
| Female | 0.590 | 0.242 | -0.365 | 0.573 | 0.245 | -0.296 | 0.033 | 0.988 |
| Age | 19.975 | 1.370 | 1.466 | 20.092 | 1.532 | 1.163 | -0.097 | 0.894 |
| *Diploma* score | 81.485 | 141.530 | 0.053 | 77.610 | 133.728 | 0.285 | 0.330 | 1.058 |
| *Type of upper secondary school* |  |  |  |  |  |  |  |  |
| *Liceo* for classical studies | 0.179 | 0.147 | 1.673 | 0.181 | 0.149 | 1.654 | -0.006 | 0.990 |
| *Liceo* for scientific studies | 0.398 | 0.240 | 0.417 | 0.351 | 0.228 | 0.624 | 0.097 | 1.051 |
| *Liceo* for other studies | 0.183 | 0.150 | 1.638 | 0.177 | 0.146 | 1.695 | 0.017 | 1.028 |
| Technical/vocational school | 0.240 | 0.182 | 1.220 | 0.291 | 0.206 | 0.922 | -0.116 | 0.883 |
| *Educational attainment* |  |  |  |  |  |  |  |  |
| *Father* |  |  |  |  |  |  |  |  |
| up to lower secondary school degree | 0.339 | 0.224 | 0.680 | 0.340 | 0.225 | 0.678 | -0.001 | 0.998 |
| upper secondary school degree | 0.451 | 0.248 | 0.196 | 0.449 | 0.248 | 0.206 | 0.005 | 1.000 |
| graduate in matched field | 0.054 | 0.051 | 3.961 | 0.073 | 0.068 | 3.276 | -0.080 | 0.747 |
| graduate not in matched field | 0.156 | 0.132 | 1.895 | 0.138 | 0.119 | 2.095 | 0.050 | 1.104 |
| *Mother* |  |  |  |  |  |  |  |  |
| up to lower secondary school degree | 0.299 | 0.210 | 0.879 | 0.274 | 0.199 | 1.011 | 0.054 | 1.052 |
| upper secondary school degree | 0.481 | 0.250 | 0.077 | 0.488 | 0.250 | 0.047 | -0.015 | 0.998 |
| graduate in matched field | 0.044 | 0.042 | 4.423 | 0.049 | 0.047 | 4.187 | -0.021 | 0.913 |
| graduate not in matched field | 0.176 | 0.145 | 1.703 | 0.188 | 0.153 | 1.594 | -0.033 | 0.947 |
| *Occupation* |  |  |  |  |  |  |  |  |
| *Father* |  |  |  |  |  |  |  |  |
| unemployed, in education | 0.094 | 0.086 | 2.775 | 0.106 | 0.095 | 2.563 | -0.038 | 0.903 |
| employee | 0.594 | 0.241 | -0.382 | 0.566 | 0.246 | -0.267 | 0.056 | 0.981 |
| self employed | 0.243 | 0.184 | 1.200 | 0.256 | 0.191 | 1.119 | -0.030 | 0.965 |
| other | 0.014 | 0.014 | 8.310 | 0.008 | 0.008 | 10.948 | 0.055 | 1.694 |
| retired | 0.055 | 0.052 | 3.895 | 0.064 | 0.060 | 3.564 | -0.037 | 0.871 |
| *Mother* |  |  |  |  |  |  |  |  |
| unemployed, in education | 0.296 | 0.208 | 0.895 | 0.267 | 0.196 | 1.051 | 0.063 | 1.062 |
| employee | 0.523 | 0.250 | -0.091 | 0.531 | 0.249 | -0.126 | -0.017 | 1.001 |
| self employed | 0.084 | 0.077 | 2.995 | 0.103 | 0.093 | 2.604 | -0.066 | 0.830 |
| other | 0.086 | 0.079 | 2.945 | 0.091 | 0.083 | 2.851 | -0.015 | 0.956 |
| retired | 0.011 | 0.011 | 9.468 | 0.007 | 0.007 | 11.847 | 0.041 | 1.540 |
| *Industry* |  |  |  |  |  |  |  |  |
| *Father* |  |  |  |  |  |  |  |  |
| business/personal services, PA | 0.326 | 0.220 | 0.741 | 0.330 | 0.221 | 0.722 | -0.008 | 0.993 |
| professional, scientific, technical activities | 0.118 | 0.104 | 2.366 | 0.110 | 0.098 | 2.485 | 0.024 | 1.059 |
| manufacturing, construction | 0.064 | 0.060 | 3.547 | 0.051 | 0.049 | 4.074 | 0.057 | 1.241 |
| other | 0.491 | 0.250 | 0.036 | 0.508 | 0.250 | -0.033 | -0.034 | 0.999 |
| *Mother* |  |  |  |  |  |  |  |  |
| business/personal services, PA | 0.238 | 0.181 | 1.230 | 0.237 | 0.181 | 1.236 | 0.002 | 1.002 |
| professional, scientific, technical activities | 0.040 | 0.039 | 4.668 | 0.033 | 0.032 | 5.268 | 0.042 | 1.230 |
| manufacturing, construction | 0.011 | 0.011 | 9.468 | 0.009 | 0.009 | 10.223 | 0.015 | 1.158 |
| other | 0.711 | 0.206 | -0.929 | 0.721 | 0.201 | -0.985 | -0.023 | 1.021 |
| *ERC sectors* |  |  |  |  |  |  |  |  |
| Social Sciences and Humanities | 0.447 | 0.247 | 0.212 | 0.501 | 0.250 | -0.005 | -0.108 | 0.988 |
| Physical Sciences and Engineering | 0.085 | 0.078 | 2.973 | 0.140 | 0.120 | 2.081 | -0.173 | 0.648 |
| Life Sciences | 0.468 | 0.249 | 0.130 | 0.359 | 0.230 | 0.586 | 0.221 | 1.081 |
| *Municipality / province of residence* |  |  |  |  |  |  |  |  |
| City of Messina | 0.278 | 0.201 | 0.993 | 0.273 | 0.199 | 1.018 | 0.010 | 1.009 |
| Other city in the province of Messina | 0.306 | 0.213 | 0.840 | 0.317 | 0.217 | 0.784 | -0.024 | 0.980 |
| Other city in the Sicilian region | 0.183 | 0.150 | 1.638 | 0.179 | 0.147 | 1.674 | 0.011 | 1.017 |
| City of Reggio Calabria | 0.100 | 0.090 | 2.668 | 0.086 | 0.079 | 2.952 | 0.048 | 1.143 |
| Other city in the province of Reggio Calabria | 0.094 | 0.086 | 2.775 | 0.099 | 0.089 | 2.688 | -0.015 | 0.959 |
| Other city in the Calabria region | 0.031 | 0.030 | 5.368 | 0.036 | 0.035 | 4.978 | -0.025 | 0.876 |
| City in another region | 0.007 | 0.007 | 11.746 | 0.009 | 0.009 | 10.223 | -0.024 | 0.764 |

*Note.* The last two columns report the standardized differences and the variance ratios, respectively, for the covariates used in the main analysis calculated by comparing the group of freshmen who earned credits in the academic year 2016-2017 with the group without credits in the same period. The variable “graduate in matched field” is a dummy variable on whether father/mother graduated in a field of study analogous to the child.

An alternative approach is to consider that the data are left-censored for GPA values strictly lower than 18, so that students who did not report exams during the year score 0. We assume that a proportion of students without grades could not be willing to take exams in any case - for example because they are not satisfied with their undergraduate experience; while for those students who are potentially engaged in education the GPA will be either 0 or positive (between 18 and 30). The double-hurdle model introduced by Cragg [3] solves the over-censoring issue by combining two steps, first estimating the students’ propensity to take exams (first hurdle) and then academic performance conditional on positive propensity (second hurdle). Using personality traits and the full set of controls as explanatory variables in the model, the estimated effects of the Big Five traits on GPA are consistent with the main results presented in Section 5 (S2 Table 5).

**S2 Table 5. Double-hurdle model of GPA scores.**

|  | (1) | (2) | (3) | (4) |
| --- | --- | --- | --- | --- |
| Extraversion | -0.115^*^ | -0.114^*^ | -0.110^*^ | -0.106^*^ |
|  | (0.048) | (0.047) | (0.046) | (0.046) |
| Agreeableness | -0.081 | -0.108^*^ | -0.112^*^ | -0.103^*^ |
|  | (0.050) | (0.049) | (0.049) | (0.049) |
| Conscientiousness | 0.289^***^ | 0.228^***^ | 0.226^***^ | 0.222^***^ |
|  | (0.050) | (0.049) | (0.049) | (0.049) |
| Emotional Stability | -0.213^***^ | -0.072 | -0.062 | -0.063 |
|  | (0.049) | (0.051) | (0.051) | (0.051) |
| Openness to experience | 0.119^*^ | 0.117^*^ | 0.119^**^ | 0.119^*^ |
|  | (0.048) | (0.046) | (0.046) | (0.046) |
| Female |  | 0.578^***^ | 0.568^***^ | 0.589^***^ |
|  |  | (0.103) | (0.104) | (0.104) |
| Age |  | -2.982^*^ | -3.262^**^ | -3.001^*^ |
|  |  | (1.240) | (1.244) | (1.247) |
| Age squared |  | 2.949^*^ | 3.234^**^ | 2.980^*^ |
|  |  | (1.239) | (1.243) | (1.245) |
| *Type of upper secondary school* |  |  |  |  |
| *Liceo* for scientific studies |  | -0.221 | -0.249 | -0.255^*^ |
|  |  | (0.128) | (0.129) | (0.129) |
| *Liceo* for other studies |  | -0.430^**^ | -0.456^**^ | -0.443^**^ |
|  |  | (0.147) | (0.151) | (0.152) |
| Technical/vocational school |  | -1.177^***^ | -1.185^***^ | -1.206^***^ |
|  |  | (0.143) | (0.149) | (0.149) |
| *ERC sectors* |  |  |  |  |
| Physical Sciences and Engineering |  | -0.052 | -0.098 | -0.135 |
|  |  | (0.172) | (0.172) | (0.172) |
| Life Sciences |  | -0.458^***^ | -0.455^***^ | -0.433^***^ |
|  |  | (0.096) | (0.096) | (0.097) |
| Parental controls |  |  | YES | YES |
| Municipality/ province of provenience |  |  |  | YES |
| Constant | 25.068^***^ | 25.394^***^ | 25.869^***^ | 25.937^***^ |
|  | (0.046) | (0.137) | (0.266) | (0.273) |
| Observations | 4102 | 4102 | 4102 | 4102 |
| Chi-square | 57.995 | 267.229 | 307.490 | 325.357 |
| *p*-value | 0.000 | 0.000 | 0.000 | 0.000 |

*Note.* The table reports estimated coefficients (with standard errors in parenthesis) from a hurdle model for the GPA score of students with a positive propensity to achievement. The omitted category of upper secondary school is the *liceo* for classical studies. The omitted category of ERC sector is Social Sciences and Humanities. Parental controls include educational attainment, occupational status and industry. *Chi-square* reports the Wald test statistic for the joint significance of the explanatory variables. Significance level: *: *p*<.05, **: *p*<.01, ***: *p*<.001.

**Personality traits and choice of the field of study**

Since the student’s choice of the course of study reflects individual aspirations, aptitudes and preferences relating to personality traits, a non-random distribution of personality profiles across degree programmes would not be surprising. To check whether the Big Five traits are conditional-balanced across fields of study, we run a series of regressions - with standard errors clustered at the course of study level - of each trait on the ERC sectors, including student and parental characteristics and dummies for the area of residence as controls. The only sources of unbalance are: (i) students in Physical Sciences and Engineering are less extraverted than students in the other two academic disciplines (*p=.029* with respect to students in Social Sciences and Humanities; *p=.007* with respect to students in Life Sciences); (ii) students in Social Sciences and Humanities are less emotionally stable than students in Life Sciences (*p=.002*). To check how these sources of unbalance affect the results, we estimate an equation including, apart from the Big Five trait scores, the full set of individual and parental controls, the ERC sectors and the interaction terms between the Big Five trait not uniformly distributed across fields of study and the corresponding ERC sector. Specifically, the interaction terms that we consider are the product of (i) extraversion by Physical Sciences and Engineering, (ii) emotional stability by Social Sciences and Humanities and (iii) emotional stability by Life Sciences. As neither the effect of extraversion on GPA for students in Physical Sciences and Engineering (or other students) nor the interaction effect between the variables are statistically significant, we conclude that this group does not drive the estimates in main analysis of Section 5. On the other hand, as emotional stability is negatively and significantly related to student performance in the Life Sciences sector (*p<.001*), we note that for this trait the effect is group-specific. Contrary to the intuition that emotional stability supports better performance outcomes, this evidence suggests that neurotic students in Life Sciences are more averse to a bad exam experience and for this reason are prone to work harder to make this an unlikely event. The results are shown in S2 Table 6.

**S2 Table 6. Estimated effects of the Big Five personality traits on GPA: heterogeneity analysis on ERC sectors.**

|  | (1) |
| --- | --- |
| Extraversion | -0.019 |
|  | (0.019) |
| Agreeableness | -0.039 |
|  | (0.022) |
| Conscientiousness | 0.093^***^ |
|  | (0.017) |
| Emotional stability | -0.015 |
|  | (0.051) |
| Openness to experience | 0.039^*^ |
|  | (0.017) |
| *ERC sectors* |  |
| Physical Sciences and Engineering | 0.064 |
|  | (0.047) |
| Life Sciences | 0.053 |
|  | (0.051) |
| Extraversion × Physical Sciences and Engineering | -0.044 |
|  | (0.054) |
| Emotional stability × Social Sciences and Humanities | 0.021 |
|  | (0.058) |
| Emotional stability × Life Sciences | -0.057 |
|  | (0.054) |
| Female | 0.135^**^ |
|  | (0.046) |
| Age | -1.079^*^ |
|  | (0.501) |
| Age squared | 1.055^*^ |
|  | (0.505) |
| *Type of upper secondary school* |  |
| *Liceo* for scientific studies | -0.102 |
|  | (0.055) |
| *Liceo* for other studies | -0.174^***^ |
|  | (0.036) |
| Technical/vocational school | -0.345^***^ |
|  | (0.059) |
| Parental controls | YES |
| Constant | 0.200 |
|  | (0.101) |
| Observations | 3242 |
| *R*^2^ | 0.051 |
| F | 124.843 |
| *p*-value | 0.000 |

*Note*. The table shows a heterogeneity analysis of the effects of extraversion and emotional stability on GPA when interactions between each Big Five trait not uniformly distributed across fields of study and the corresponding ERC sector are included in the regression equation. The omitted category of upper secondary school is the *liceo* for classical studies. The omitted category of ERC sector is Social Sciences and Humanities. Parental controls include educational attainment, occupational status and industry. The estimated effect of emotional stability for students in Life Sciences is negative and statistically significant (*β= -.072,* *p<.01*). Significance level (*: *p*<.05, **: *p*<.01, ***: *p*<.001) based on robust standard errors (reported in parenthesis), clustered at the course of study level (46 clusters).

Further, we consider whether the effects of openness to experience differ across fields of study. We include the full set of interaction terms between openness to experience and the ERC sectors in a regression of academic performance on the Big Five trait scores and the set of predetermined characteristics. The results indicate that the effects of openness to experience are not heterogeneous across fields of study (S2 Table 7).

**S2 Table 7. Estimated effects of the Big Five personality traits on GPA: openness to experience and ERC sectors.**

|  | (1) |
| --- | --- |
| Extraversion | -0.024 |
|  | (0.019) |
| Agreeableness | -0.040 |
|  | (0.023) |
| Conscientiousness | 0.093^***^ |
|  | (0.017) |
| Emotional Stability | -0.030 |
|  | (0.023) |
| Openness to experience | 0.052^*^ |
|  | (0.022) |
| *ERC sectors* |  |
| Physical Sciences and Engineering | 0.080 |
|  | (0.050) |
| Life Sciences | 0.054 |
|  | (0.054) |
| Openness to experience × Physical Sciences and Engineering | 0.028 |
|  | (0.060) |
| Openness to experience × Life Sciences | -0.038 |
|  | (0.042) |
| Female | 0.138^**^ |
|  | (0.046) |
| Age | -1.078^*^ |
|  | (0.488) |
| Age squared | 1.054^*^ |
|  | (0.493) |
| *Type of upper secondary school* |  |
| *Liceo* for scientific studies | -0.102 |
|  | (0.055) |
| *Liceo* for other studies | -0.175^***^ |
|  | (0.036) |
| Technical/vocational school | -0.348^***^ |
|  | (0.059) |
| Parental controls | YES |
| Constant | 0.200 |
|  | (0.101) |
| Observations | 3242 |
| *R*^2^ | 0.050 |
| F | 88.122 |
| *p*-value | 0.000 |

*Note.* The table shows the interaction effects of openness to experience on GPA across fields of study. The omitted category of: (i) upper secondary school is the *liceo* for classical studies; (ii) ERC sector is Social Sciences and Humanities. The interaction term between openness to experience and the sector Social Sciences and Humanities is omitted. Parental controls include educational attainment, occupational status and industry. The postestimation test of equality in the coefficients for the interaction terms included in the regression fails to reject the null hypothesis (*Prob*>F=.324). Significance level (*: *p*<.05, **: *p*<.01, ***: *p*<.001) based on robust standard errors (reported in parenthesis), clustered at the course of study level (46 clusters).

**Selection on unobservables**

As explained in Section 4, the estimation strategy of this study relies on the fulfilment of the conditional independence assumption and, therefore, on the limitation of the bias due to omitted confounders. In this view, one evaluates the stability of the coefficients after the inclusion of controls in the equation. In theory, this procedure may not be able to warrant consistent estimates against the omitted variables problem and this risk is higher when the observed variables explain only a small fraction of the variance of the outcome variable. To discard further concerns about selection effects in the distribution of personality traits across study programmes and to account for the influence of other potential unobservable confounders, we consider the strategy introduced by Altonji et al. [4]. The authors propose a methodology to estimate the ratio of selection on unobservables relative to selection on observables that would be required for the treatment effect to be entirely due to omitted variable bias. The rationale of this test is that the sensitivity of the estimated coefficients to the inclusion of controls is not a sufficient diagnostic of the omitted variable bias. Coefficient changes are proportional to the omitted variable bias only if they are scaled by changes in the fraction of explained variance when the control variables are included [5].

On this basis, we consider each dimension of personality separately to test its impact on student performance by calculating an estimator of the coefficient of proportionality (*delta*) under the null hypothesis that the treatment effect is not significantly different from zero. The empirical setup is straightforward and draws directly from the regression model (1). The *j^th^* personality trait is the variable of interest, which relates to a vector of observed characteristics –personality traits are allowed to correlate with each other – and an index of unobservable confounders, with the observed controls and the unobservables being mutually orthogonal.

This paper presents two tests for selection on unobservables. The first test follows the empirical approach introduced by Bellows and Miguel [6] and its application in Nunn and Wantchekon [7]. Bellows and Miguel [6] build up an estimator of the coefficient of proportionality in the case of a linear regression model with a continuous treatment variable. Their approach diverges from the methodology of Altonji et al. [4] in that they implicitly assume that observed and unobserved controls are equal in variance [8]. We distinguish between *^R^*, the estimated coefficient from a restricted regression equation of the *j^th^* factor score on the dependent variable, and the estimated coefficient *^F^* from a regression that includes the full set of control variables (specification IV of Table 4). Hence, the coefficient of proportionality *δ* is calculated as follows: *^F^/(^R^-^F^)*. A formal description of the ratio is provided in the Appendix of Bellows and Miguel [6]. Oster [5] formalises the assumptions of the model and develops a consistent estimator of the omitted variable bias. The greater is the magnitude of *^F^*, the larger is the effect that needs to be explained by selection on unobservables. The smaller are the coefficient movements in the denominator, the lower is the selection on observables and the greater should be the selection on unobservables to explain away the observed effect.

The second test follows the approach recently employed by Oster [5], i.e. to set a value for R-squared in the case that the full set of controls (observable and unobservable variables) were included in the model, *R_max_*, and then to calculate the value of the coefficient of proportionality *δ* assuming the treatment effect equal to zero. Oster posits that the approach of Altonji et al. [4] is restrictive in assuming that, if the unobservable factors were included in the regression model, the R-squared coefficient would be equal to 1, since it does not take into account possible sources of exogenous variation in the outcome such as unobserved factors which are not predetermined characteristics or measurement error. Thus, the bounding value of *R_max_* is set to 1.3 times the R-squared coefficient from a regression which includes the complete set of observed controls, . Oster calculates the multiplier as the cut-off value that would allow at least 90 per cent of the randomized controlled trials published in the five top journals between 2008 and 2013 to survive the selection adjustment criteria (for nonrandomized data the survive rate drops to 45 per cent). Again, the proportional selection relationship depends on a comparison between the univariate (uncontrolled) regression of Y on the *j^th^* personality trait of interest and the full regression model.

The results are summarized in S2 Table 8. The *δ* values reported in column 1 are computed using the Bellows and Miguel formula, while in column 2 follow the Oster procedure. All the ratios are greater than one in absolute value and generally robust to validation analysis. To the extent that selection on observables and selection on unobservables are positively correlated, a delta value showing a negative sign means that, if anything, the estimated effect of the treatment variable is downward biased by selection on unobservables. The estimated effects of conscientiousness and openness to experience appear robust to the selection adjustment. Doubling the value of *R_max_* in the Oster formula, the selection on unobservables should be 3.239 times more important than selection on observables to capture the entire effect of conscientiousness on student performance (the delta ratio is -2.963 with respect to openness to experience).

**S2 Table 8. Amount of selection on unobservables relative to selection on observables for *β=0*.**

|  |  | δ ratio for *β=0* | |  |
| --- | --- | --- | --- | --- |
|  | Treatment variable | Bellows and Miguel  (2009) | Oster  (2019) |  |
|  | Extraversion | 10.300 | 13.155 |  |
|  | Agreeableness | -1.247 | -4.0572 |  |
|  | Conscientiousness | -15.625 | 13.222 |  |
|  | Emotional stability | 5.234 | 5.378 |  |
|  | Openness to experience | -3.606 | -14.872 |  |

*Note*. The table shows the amount of selection on unobservables relative to selection on observables to explain away the entire treatment effect. In column 1, we use the Bellows and Miguel method, which satisfies the condition that observed and unobserved controls equally explain the variance of the outcome. In column 2, we use the Oster formula and set the upper bound on *R_max_* equal to 1.3 times the R-squared from a regression equation that includes the entire set of observed controls.

**References**

1. Normand SLT, Landrum MB, Guadagnoli E, Ayanian JZ, Ryan TJ, Cleary PD, et al. Validating recommendations for coronary angiography following acute myocardial infarction in the elderly: a matched analysis using propensity scores. J Clin Epidemiol. 2001;54: 387–398. doi:10.1016/S0895-4356(00)00321-8

2. Rubin DB. Using propensity scores to help design observational studies: application to the tobacco litigation. Heal Serv Outcomes Res Methodol. 2001;2: 169–188. doi:10.1023/A:1020363010465

3. Cragg JG. Some statistical models for limited dependent variables with application to the demand for durable goods. Econometrica. 1971;39: 829–844. doi:10.2307/1909582

4. Altonji JG, Elder TE, Taber CR. Selection on observed and unobserved variables: assessing the effectiveness of Catholic schools. J Polit Econ. 2005;113: 151–184. doi:10.1086/426036

5. Oster E. Unobservable selection and coefficient stability: theory and evidence. J Bus Econ Stat. 2019;37: 187–204. doi:10.1080/07350015.2016.1227711

6. Bellows J, Miguel E. War and local collective action in Sierra Leone. J Public Econ. 2009;93: 1144–1157. doi:10.1016/j.jpubeco.2009.07.012

7. Nunn N, Wantchekon L. The slave trade and the origins of mistrust in Africa. Am Econ Rev. 2011;101: 3221–3252. doi:10.1257/aer.101.7.3221

8. González F, Miguel E. War and local collective action in Sierra Leone: a comment on the use of coefficient stability approaches. J Public Econ. 2015;128: 30–33. doi:10.1016/j.jpubeco.2015.05.004
